# Epigenetic Gene Regulation by Dietary Compounds in Cancer Prevention

**DOI:** 10.1093/advances/nmz046

**Published:** 2019-05-17

**Authors:** McKale Montgomery, Aishwarya Srinivasan

**Affiliations:** Department of Nutritional Sciences, Oklahoma State University, Stillwater, OK

**Keywords:** DNA methylation, histone modifications, noncoding RNAs, chemoprevention, bioactive component

## Abstract

Traditionally, cancer has been viewed as a set of diseases that are driven by the accumulation of genetic mutations, but we now understand that disruptions in epigenetic regulatory mechanisms are prevalent in cancer as well. Unlike genetic mutations, however, epigenetic alterations are reversible, making them desirable therapeutic targets. The potential for diet, and bioactive dietary components, to target epigenetic pathways in cancer is now widely appreciated, but our understanding of how to utilize these compounds for effective chemopreventive strategies in humans is in its infancy. This review provides a brief overview of epigenetic regulation and the clinical applications of epigenetics in cancer. It then describes the capacity for dietary components to contribute to epigenetic regulation, with a focus on the efficacy of dietary epigenetic regulators as secondary cancer prevention strategies in humans. Lastly, it discusses the necessary precautions and challenges that will need to be overcome before the chemopreventive power of dietary-based intervention strategies can be fully harnessed.

## Introduction

Dietary factors are second only to tobacco as preventable causes of cancer in Western countries (1). Both micronutrient insufficiencies and macronutrient excess are known contributors to cancer development and progression, yet worldwide micronutrient deficiencies persist, and obesity rates are at an all-time high (2). As such, alternative diet-based chemopreventive approaches are fervently being sought. The term “chemoprevention” was first used in 1976 in the context of work with vitamin A and retinoids, and defined as “the use of natural or synthetic agents to block, retard, or reverse the carcinogenic process” (3). Thus, the idea of utilizing dietary components to prevent cancer development is not a new concept, but our understanding of their chemoprotective actions is rapidly evolving.

Epigenetics is defined as heritable modifications to the genome that do not involve a change in DNA sequence. By influencing gene expression of the individual, epigenetic modifications determine human appearance, behavior, stress response, disease susceptibility, and even longevity, giving rise to the individual phenotype. As such, epigenetic mechanisms are essential for regulating normal physiologic processes, and aberrant epigenetic alterations have been implicated in the pathology of numerous diseases. Unlike genetic inheritance, epigenetic marks are influenced by things such as lifestyle, environment, and nutritional status. Thus, targeting the epigenome to treat and prevent disease is a promising therapeutic approach. Epigenetic control of gene expression is mediated via DNA methylation, histone modifications, and noncoding RNAs, and importantly each of these control points can be targeted by dietary components.

## Current Status of Knowledge

### Part 1: overview of epigenetic regulation

#### DNA methylation

DNA methylation involves the covalent transfer of a methyl group to DNA by DNA methyltransferases (DNMTs) (4). Most DNA methylation occurs within a region in which a cytosine nucleotide is attached to a guanine nucleotide via a phosphate linkage, which is known as a CpG site (5). Dense repeats of CpG nucleotides, called CpG islands, occur throughout the genome, although the majority of methylated CpG islands are associated within protein-coding genes (4). Methylation of CpG islands within the promoter region of a gene is typically inversely associated with transcription of that gene due to binding of methyl-CpG binding proteins, which subsequently block transcription (6). During cellular replication, DNA methylation patterns are maintained and passed on from the parental strand of DNA via the enzymatic action of DNMT1 (7). In contrast, DNMT3A and DNMT3B are referred to as de novo methyltransferases because of their ability to produce new DNA methylation marks within CpG dinucleotides, which are especially important in early development (6). A classic example of DNA methylation and epigenetic regulation is the diet-modified phenotype of the agouti gene, which regulates coat color and weight in mice. When the gene is unmethylated, and thus actively being transcribed, the resulting phenotype is an obese mouse with a yellow coat. However, this activation can be suppressed by promoting DNA methylation via a methyl-rich diet. Importantly, maternal supplementation with a methyl-rich diet is sufficient to repress agouti overexpression in offspring as well (8). Changes in both global and gene-specific DNA methylation patterns can influence cancer development.

#### Histone modifications

Histones are the primary components of chromatin, the DNA-protein complex that makes up chromosomes. Within the nucleus, DNA winds tightly around an octamer of histones, and as such histone modifications can influence chromatin arrangement and DNA transcription (9). Histones can be modified by acetylation, methylation, phosphorylation, ubiquitination, ADP-ribosylation, and biotinylation of their N-terminal histone tails (6).

Histone acetylation is conferred by histone acetyltransferases (HATs), which transfer acetyl groups onto the ε-amino group of a lysine residue within the histone tail. Subsequently, the charge of the lysine is neutralized and the interaction between the histone tail and DNA is weakened, leading to chromatin relaxation, and gene transcription (10). In contrast to HATs, histone deacetylases (HDACs) remove acetyl groups from lysines and restore the positive charge on the histone tail, and are generally thought of as transcriptional repressors. Histone phosphorylation and dephosphorylation of serines, threonines, and tyrosines within histone tails is mediated by histone kinases and phosphatases, respectively (11). Like acetylation, histone phosphorylation also alters the charge of the histone protein, thereby altering the structure of the chromatin environment (6). Methylation, on the other hand, does not change the ionic charge of the histone protein. Rather, methylation of lysine and arginine residues within histone tails influences gene transcription through the recruitment and binding of effector molecules (11). Histone ubiquitination is less well understood than the other histone modifications, but we do know that it is tightly regulated by specific histone ubiquitin ligases and deubiquitinating enzymes. Moreover, although many proteins are targeted for ubiquitination, histones are by far the most ubiquitinated proteins in the nucleus, and this helps them perform critical roles including transcription, maintenance of chromatin structure, and DNA repair (12). As such, aberrant histone modifications have been implicated in all stages of cancer development.

#### Noncoding RNAs

Epigenetic control can also be regulated via noncoding RNA (ncRNA)-based mechanisms. Generally, ncRNAs are subdivided based on size into long (>200 nt) or small ncRNAs. Small ncRNAs are also further categorized into microRNAs (miRNAs), small interfering RNAs or PIWI-interacting RNAs. Thousands of miRNA and long noncoding (lncRNAs) are encoded within the human genome, and are often expressed in a cell-type-, tissue-, and disease-specific manner (13). Together, these classes of RNA species make up the more than two-thirds of the human genome that is transcribed but not translated into proteins, although each play significant roles in regulating the expression and function of protein-coding genes. To this end, the epigenetic nature of miRNA regulation is reciprocal in nature. miRNA transcription can be modulated by both DNA methylation and histone modifications, and miRNA themselves can, in turn, regulate crucial enzymes that drive epigenetic remodeling (14–17).

To regulate gene expression miRNA must first assemble into a multiprotein RNA-induced silencing complex (RISC). Once assembled, the bound miRNA/RISC complex is then competent to target a given mRNA based on the recognition of target sequences within a given mRNA. The bound miRNA/RISC complex negatively regulates target gene expression via transcript degradation or translational inhibition, or a combination of both (18). lncRNAs, on the other hand, may regulate gene expression thoug

h multiple mechanisms: by functioning as signals for transcription initiation, by acting as decoys for titrating transcription factors and miRNA, by serving as guides for chromatin-modifying enzymes, or by serving as scaffolds for the formation of ribonuecleoprotein complexes (19, 20). Because of their dynamic expression and functional versatility, ncRNAs have been demonstrated to contribute to a number of critical physiologic processes, and their dysregulation has been implicated in the pathogenesis of many disease states (21). With regards to human cancer development and prevention, miRNA and lncRNAs are the best-characterized ncRNAs, with each having established oncogenic and tumor-suppressive functions (22–24).

### Part 2: dietary epigenetic regulators in cancer prevention

Cancer risk, and epigenetic markers such as DNA methylation and histone acetylation, are shaped by both genetic predisposition and environmental influences. As such, epigenetic markers can provide critical etiologic insight into how genetic code is translated into biological action, and thus epigenetic-based therapies provide opportunities for the development of precision medicine. Indeed, epigenetic biomarkers have demonstrated utility in cancer risk prediction, diagnostics, treatment, and even predicting the treatment response (25–27). Once cancer has developed, however, the genetic diversity and complexity of many cancers often renders treatments ineffective. Thus, identifying effective strategies for chemoprevention is necessary for reducing the global burden of cancer.

**FIGURE 1:**
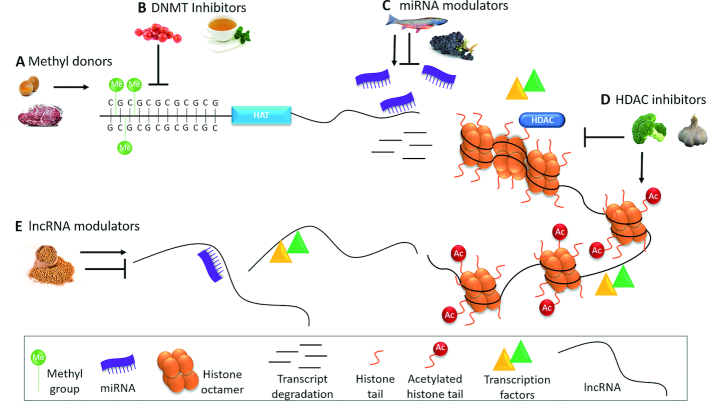
Overview of the complexity and overlap of diet-based epigenetic regulatory mechanisms. Bioactive components of dietary sources can alter DNA methylation by (A) serving as methyl donors for DNA methylation, or (B) preventing DNA methylation by acting as DNMT inhibitors. Decreased DNA methylation promotes transcription of genes, such as HATs. (C) Dietary miRNA modulators can either upregulate or downregulate miRNA expression. miRNA controls gene expression by binding to target mRNAs and subjecting them to translational repression or transcript degradation. Degradation of HAT transcripts would decrease histone acetylation, resulting in transcriptional repression via chromatin compaction. (D) By preventing histone deacetylation, dietary HDAC inhibitors can promote histone acetylation and chromatin relaxation, thereby making DNA more accessible to transcription factors. (E) Dietary components can also modulate the transcription of lncRNAs, which can then influence gene expression by acting as decoys for miRNA and transcription factors. DNMT, DNA methyltransferase; HAT, histone acetyltransferase; HDAC, histone deacetylase; lncRNA, long noncoding RNA; miRNA, microRNA.

Chemoprevention can be broadly defined to include a range of approaches such as avoidance of carcinogen exposure (primary prevention), blocking, slowing, or reversing cancer progression (secondary prevention), and subduing or removing precancerous lesions (tertiary prevention). The reversible nature of epigenetic modifications makes them desirable targets for chemoprevention. Interestingly, bioactive components from both essential and nonessential dietary compounds can act as epigenetic regulators by influencing DNA methylation, histone modifications, and ncRNA expression and function (**Figure 1**). It is not surprising, then, that bioactive components from dietary sources have been suggested to have efficacy in primary, secondary, and tertiary cancer prevention strategies.

Harnessing the chemopreventive power of such dietary agents is complicated, however, because they can be metabolized into many unique bioactive metabolites, which often have overlapping impacts on epigenetic control mechanisms. For example, glycosinolates, which are found in cruciferous vegetables, can be broken down into isothiocyanate (sulforaphane), phenethyl isothiocyanate (PEITC), indole-3-carbinol, and 3,3′-diindolylmethane—all of which are chemopreventive, and each of which can influence DNA methylation, histone modifications, and miRNA expression (28). Furthermore, to elicit these epigenetic alterations, and exert its chemopreventive actions, the resultant bioactive metabolite has to first enter circulation at sufficient concentrations such that it can actually reach its target tissue. Thus, the effectiveness of a given dietary compound is dependent upon the bioavailability of the bioactive component. Bioavailability, and subsequent efficacy, are, however also affected by the intrinsic genetic, epigenetic, and environmental influences of the individual. The mixed results of the preclinical and clinical studies described below further highlight the complexity of developing population-level dietary intervention chemopreventive strategies.

#### Chemopreventive potential of dietary DNMT inhibitors

Variations in the degree or site of DNA methylation can lead to disruption of chemoprotective cellular processing leading to tumor initiation and progression. Indeed, aberrant DNA methylation patterns are hallmarks of many types of cancers. For example, global hypomethylation is linked to chromosomal instability, whereas promoter hypermethylation is associated with gene silencing of tumor suppressors in cancers (29, 30). Substantial evidence suggests that the anticancer properties of many bioactive food components may, at least in part, be attributed to their capacity to influence DNA methylation patterns. Deficiencies in zinc and selenium, as well as excess retinoic acid, have been shown to lead to global hypomethylation, and are associated with increased cancer risk (30). Dietary components can also influence DNA methylation patterns by providing substrates and acting as cofactors that are necessary for 1-carbon metabolism. The availability of the universal methyl donor, *S*-adenosylmethionine is determined by 1-carbon metabolism, and is critical for proper DNA and histone methylation control. Nutrients involved in the 1-carbon metabolism pathway include vitamins B-6, B-12, folate, riboflavin, betaine, and choline, as well as the amino acids methionine, cysteine, serine, and glycine (6). Dietary insufficiencies in any one of these nutrients can lead to global DNA hypomethylation, via disruption of this pathway (30).

Dietary agents can also influence the enzymatic activities of DNMTs (30). As promoter hypermethylation of tumor suppressor genes is common in many cancers, DNMT inhibitors are promising agents for epigenetic therapy. Two synthetic DNMT inhibitors, azacytidine and decitabine, are already FDA approved for the treatment of myelodysplastic syndrome and acute myeloid leukemia (26). However, the pleiotropic molecular effects and systemic toxicity events associated with pharmacologic DNMT inhibitors precludes their use as a primary preventative strategy in healthy individua

ls. Thus, the identification of diet-derived DNMT inhibitors and their efficacy as chemopreventive agents has received much attention.

Dietary polyphenols, particularly (–)-epigallocatechin 3-gallate (EGCG) from green tea, and genistein, a soy isoflavone, are perhaps the most well-studied dietary DNMT inhibitors, although many others have also been identified (**Table 1**). EGCG and genistein exert their anticancer activity via direct inhibition of DNMT1, which reactivates methylation-silenced tumor suppressors such as *CDKN2A* and *O*^6^-methylguanine-DNA methyltransferase (31, 32). Both EGCG and genistein have been demonstrated to effectively deter carcinogenesis in animal models (33, 34). However, epidemiologic data regarding the anticancer properties of EGCG and genistein in humans has been mixed (35, 36). Unfortunately, early-phase clinical trials have not yielded much more promising results.

**TABLE 1:** Chemopreventive actions of dietary DNMT inhibitors^1^

Bioactive component	Source	Target	Anticancer effects	Type of cancer	Model system	Reference
Apigenin	Fruits and vegetables	*NFE2L, DNMT1, DNMT3A*,	↓Viability	Skin cancer	Cell lines	31, 42
Curcumin	Turmeric	*DNMT1, CDKN2B, NEUROG1, NFE2L2*	↓Proliferation ↑Apoptosis	Acute myeloid leukemia, prostate cancer	Cell lines, mouse xenografts	43–45
Daidzein	Soy	*BRCA1, GSTP1, EPHB2*	↓Proliferation	Prostate cancer	Cell lines	46, 47
EGCG	Green tea	*RECK, CDKN2A, TERT*	↓Invasiveness ↓Proliferation ↑Apoptosis	Squamous cell carcinoma, colon cancer, breast cancer	Cell lines	48–50
Genistein	Soy	*GSTP1, CDKN1A, RARB, CDKN2A MGMT, BTG3*	↓Proliferation ↓Tumorigenesis	Breast cancer, prostate cancer	Cell lines, human prostatectomies	51–54
Lycopene	Tomatoes	*GSTP1*	↓Proliferation	Breast cancer	Cell lines	52
Resveratrol	Stilbenes	*DNMT3B, PTEN*	↓Proliferation	Breast cancer	ACI rats, cell lines	55, 56
Sulforaphane	Cruciferous vegetables	*NFE2L2, TERT, DNMT1, DNMT3A*	↓Proliferation ↑Apoptosis	Prostate cancer, breast cancer	Cell lines	57–59

1 *BRCA1*, BRCA1 DNA repair associated; *BTG3*, BTG antiproliferation factor 3; *CDKN1A*, cyclin-dependent kinase inhibitor 1A; *CDKN2A*, cyclin-dependent kinase inhibitor 2A; *CDKN2B*, cyclin-dependent kinase inhibitor 2B; *DNMT1*, DNA methyltransferase 1; *DNMT3A*, DNA methyltransferase 3A; *DNMT3B*, DNA methyltransferase 3B; *EGCG*, (–)-epigallocatechin 3-gallate; *EPHB2*, EPH receptor B2; *GSTP1*, glutathione S-transferase π 1; *MGMT, O*^6^-methylguanine-DNA methyltransferase; *NFE2L2*, nuclear factor, erythroid 2–like 2; *PTEN*, phosphatase and tensin homolog; *RARB*, retinoic acid receptor β; *RECK*, reversion-inducing cysteine-rich protein with kazal motifs; TERT telomerase reverse transcriptase.

In a randomized, placebo-controlled study, daily intake of 400 mg EGCG did not reduce the likelihood of prostate cancer in men with high-grade prostatic intraepithelial neoplasia or atypical small acinar proliferation (or a combination of both) (37). Similarly, a 4-mo intervention trial with resveratrol, which also has DNMT inhibitor properties, did not reduce prostate size and concentrations of prostat

e-specific antigen (PSA) in men with metabolic syndrome (38). The highest dose of resveratrol (1000 mg) did significantly decrease serum concentrations of androgen precursors, however, suggesting a lengthier intervention time may have had a more positive impact (38). Conversely, a randomized trial of soy isoflavone supplementation not only did not reduce breast cancer risk in women, but it increased breast epithelial proliferation in premenopausal women (39). The suggestion that soy exposure may be more beneficial earlier in life could help explain these null and somewhat conflicting findings (40). Moreover, none of the aforementioned studies measured the impact of their dietary interventions on epigenetic marks, and it is therefore difficult to draw conclusions regarding their effectiveness as epigenetic regulators in this regard. Although it is worth noting that secondary and tertiary prostate cancer prevention efforts with genistein, as well as other dietary DNMT inhibitors such as curcumin, catechin, epicatechin, lycopene, and quercetin, have yielded some more promising clinical outcomes (41). One reason intervention trials may not support epidemiologic studies is because intervention trials often administer single, high doses, which do not mimic the small amounts of bioactive components that people consume daily as part of a mixed diet. Future research should assess dietary patterns rather than single dietary components, paying particular attention to how timing of dosing might influence bioavailability and efficacy.

In addition, many cancers have a very long latency period, thus the intervention in the trials described above may have occurred too late in the cancer continuum, and early-life interventions may be more effective. Epidemiologic data suggest that adult disease risk is associated with nutrient exposures early in life, and findings from the Dutch Hunger Winger studies have demonstrated the importance of epigenetic imprinting in these lifelong phenotypic consequences (60). Maternal obesity and in utero epigenetic reprogramming are also associated with increased risk of some cancers, particularly breast and colon cancers (61). Paternal obesity can also negatively affect offspring insulin-like growth factor 2 (*IGF2*) methylation, and these types of epigenetic markers can persist throughout their lifetime (61, 62). In a recent study, however, dietary supplementation with DHA during pregnancy could potentially modulate some of the adverse effects of maternal overweight and obesity by influencing *IGF2* methylation (63). Thus, dietary-based epigenetic cancer prevention needs to be thought of not just on the scale of the cancer continuum, but along the continuum of a lifespan.

In addition to bioavailability, dosing, and timing of exposure to potential dietary chemopreventive agents, the existing DNA methylation patterns of the individual may also influence the response to a bioactive food component (30). For example, pretreatment with the pharmacologic DNMT inhibitor, decitabine, increases 1,25-dihydroxycholecalciferol-induced differentiation in several mixed-lineage leukemia cell lines (64). DNA methylation status can also affect the cellular response to HDAC inhibitor treatment, indicating a reciprocal relation exists between the epigenome of the individual and the epigenetic efficacy of bioactive dietary components (65). Therefore, it is important to consider the influence of a given bioactive dietary component within the context of the entire diet.

#### Chemopreventive potential of dietary HDAC inhibitors

Posttranslational modifications of histones are critical for controlling many cellular processes, such as gene expression, as well as DNA replication and repair, and thus aberrant histone modifications have been linked to each stage of carcinogenesis. Indeed, of the >60 different histone residues in which modifications have been described, many have now been linked to cancer (98). Because of the signi

ficant contribution of these so-called histone “onco-modifications” to the hallmarks of cancer, HDAC inhibitors have been sought after for their clinical utility. Four HDAC inhibitors are already FDA approved for the treatments of lymphoma and multiple myeloma. However, their pleiotropic impact on gene expression, and lack of efficacy in solid tumors has led to the pursuit of novel HDAC inhibitors and their utility in chemoprevention instead of chemotherapy. Many dietary HDAC inhibitors have now been identified, and their chemotherapeutic and chemopreventive efficacy has been established both in vitro and in animal models (**Table 2**). So far evidence of their chemoprotective efficacy in humans is limiting, but some early stage clinical trials are promising.

**TABLE 2:** Chemopreventive actions of dietary HDAC inhibitors^1^

Bioactive component	Source	Target	Anticancer effects	Type of cancer	Model system	Reference
Allicin, allyl mercaptan, diallyl disulfide	Garlic	*CDKN1A*	↓Proliferation ↓Angiogenesis	Colon cancer, erythroleukemia, liver cancer, prostate cancer	Cell lines	66–69
Apigenin	Fruits and vegetables	C*DKN1A*	↑Apoptosis ↓Proliferation	Prostate cancer	Cell lines, mouse xenografts	42, 70
Butyrate	Soluble fibers	*CDKN1A*	↑Apoptosis ↓Proliferation	Colon cancer	Cell lines, rat carcinogen–induced colon cancer	71–74
Curcumin	Turmeric	*DLEC1, NFKB1*	↑Apoptosis ↓Proliferation ↓Tumorigenesis	Colon cancer, leukemia	Cell lines	75–77
Daidzein and genistein	Soy	*CDKN1A, CDKN2A,ESR2, BTG3*	↓Proliferation	Prostate cancer, renal cancer	Cell lines	53, 54, 78
EGCG	Green tea	*GSTP1, CDKN1A, CDKN2A*	↓Proliferation	Cervical cancer, prostate cancer, skin cancer, breast cancer	Cell lines	79–82
Indole-3 carbinol diindolylmethane	Cruciferous vegetables	*CDKN1, CDKN1B*	↓Inflammation ↑Apoptosis ↓Proliferation	Colon cancer, prostate cancer, breast cancer	Cell lines, mouse xenografts	83–85
Piceatannol	Berries, red grapes	*HDAC4, HDAC5*	↑Apoptosis ↓Proliferation ↓Inflammation	Multiple types	Renal fibrosis mouse model, cell lines	86, 87
Quercetin	Apples, dark cherries, berries	*SIRT1, FASLG*	↑Apoptosis ↓Proliferation ↓Angiogenesis ↓Invasiveness	Hepatocellular carcinoma, leukemia	Cell lines, hamster buccal pouch tumors	88–90
Resveratrol	Stilbenes	*TP53, SIRT1*	↑Apoptosis ↓Proliferation	Prostate cancer, hepatoblastoma	Cell lines	91–93
Sulforaphane	Cruciferous vegetables	*CDKN1A, TERT,DEFB4A*	↑Apoptosis ↓Proliferation ↑Immune reponse	Prostate cancer, colorectal cancer, breast cancer	Cell lines, mouse xenografts, human subjects	57, 94–97

1 *BTG3*, BTG antiproliferation factor 3; *CDKN1A*, cyclin-dependent kinase inhibitor 1A; *CDKN2A*, cyclin-dependent kinase inhibitor 2A; *DEFB4A*, defensin β 4A; *DLEC1*, DLEC1 cilia- and flagella-associated protein; *EGCG*, (–)-epigallocatechin 3-gallate; *ESR2*, estrogen receptor 2; *FASLG*, Fas ligand; *GSTP1*, glutathione *S*-transferase π 1; *HDAC4*, histone deacetylase 4; *HDAC5*, histone deacetylase 5; *NFKB1*, nuclear factor κB subunit 1; *SIRT1*, sitruin 1; *TERT*, telomere reverse transcriptase; *TP53*, tumor protein 53.

Allyl derivatives from garlic have been shown to induce histone acetylation in various human cancer cells. The most potent allyl derivative with regards to HDAC inhibition is allyl mercaptan, which exerts its anticancer properties in vitro via the hyperacetylation of *CDKN1A*, which subsequently increases *CDKN1A* gene expression and promotes cell cycle arrest (66). In preclinical studies the reported mechanisms of action of garlic-derived compounds for cancer prevention and treatment are much more diverse, and range from inducing apoptosis and autophagy to inhibiting angiogenesis and proliferation (99, 100). A randomized crossover feeding trial in humans demonstrated that a single meal of raw, crushed garlic influences the expression of multiple immunity- and cancer-related genes, suggesting the bioactivity of garlic is multifaceted (101). However, in a randomized, double-blind clinical intervention study, 7 y of garlic supplementation did not reduce the incidence of precancerous gastric lesions or gastric cancer in subjects at high risk for gastric cancer (102). This could potentially be explained because the population group was already high risk for gastric cancer, but the widespread utility of garlic supplementation will likely not be able to be utilized until the mechanisms of action are more fully understood.

Dietary isothiocyanates have also been shown to mediate anticancer activities via their HDAC inhibitory properties (103). Isothiocyanates, such as sulforaphane, are the biologically active derivatives of glucosinolates, which are abundant in cruciferous vegetables. In preclinical studies sulforaphane has been reported to induce DNA damage in colon cancer cells, and to inhibit tumor growth in mice (104, 105). In humans, increased cruciferous vegetable consumption has been associated with decreased risk of cancer development, likely via HDAC inhibition (106). In an evaluation of baseline data of women who had abnormal mammogram findings and were scheduled for breast biopsy, total cruciferous vegetable intake was associated with decreased cell proliferation in breast ductal carcinoma in situ tissue (107). This same cohort of women was then randomized in a double-blind controlled trial to consume a placebo or a 250 mg broccoli seed extract 3 times/d for 2–8 wk (108). Although circulating sulforaphane metabolites were statistically increased in the treatment group compared with the placebo, supplementation did not produce measurable changes in breast tissue biomarkers (108). In a similar study investigating the chemopreventive potential of sulforaphane in men, supplementation with 200 µmol/d of sulforaphane-rich extracts for 20 wk did not reduce PSA by ≥50%, which was the primary endpoint of the study (109). The study designs make it difficult to determine whether the negative results were because of insufficient dosing or insufficient duration, or both, so future studies will be needed to determine if dietary sulforaphane regimens might be useful chemoprevention strategies.

Additionally, the discrepancies observed between epidemiologic data of cruciferous vegetable intake and sulforaphane supplementation may also be attributed to differences in bioavailability. Sulforaphane is formed by the hydrolysis of its glucosinolate precursor, glucophanin, by the plant enzyme myrosinase, which is activated by damage to the plant tissue that occurs during chewing (110). Sulforaphane absorption is lower in adults consuming glucoraphanin supplements than fresh broccoli sprouts, but this can be improved when the supplements are consumed with a source of active myrosinase (111, 112). Treatment of glyophanin-rich broccoli extracts with myrosinase prior to supplementation has also been demonstrated to enhance sulforaphane bioavailability (113). Furthermore, a recent study also reported that subjects consuming two 100-µmol doses of sulforaphane containing broccoli extract 12 h apart retained higher plasma sulforaphane metabolite concentrations than subjects consuming one 200-µmol dose every 24 h (110).

Unfortunately, although most data support the use of whole-food strategies in dietary chemoprevention efforts, limitations in availability, and variations in bioactive content of whole-food sources often necessitate the use of supplements in clinical trials to deliver consistent doses of the bioactive components. The findings described above, however, highlight the importance of considering both the source and the dosing regimen of dietary supplements in the development of effective chemoprevention strategies. To be an effective chemopreventive agent, sufficient concentrations of the bioactive compounds must actually reach the target tissue. In the case of curcumin, which also exhibits HDAC inhibitory properties, but poor oral bioavailability, investigators have also explored nanoformulations, bioenhancers, and synthetic analogs to increase its solubility and stability and improve delivery to target tissues (114). Promising results with synthetic analogs, such as increased concentrations of bioactive curcumin metabolites in target tissues, warrant further investigation into their chemopreventive efficacy.

Although many challenges remain to be overcome, the powerful epigenetic regulatory capacity of dietary HDAC inhibitors underscores their promising chemopreventive potential. By targeting histones, HDAC inhibitor treatment influences chromatin structure and affects gene expression at many levels, and thus HDAC inhibitors can influence many diverse cellular functions, such as inducing apoptosis, disrupting cellular growth and differentiation, and inhibiting angiogenesis (Table 2). Nonhistone proteins, such as transcription factors and metabolic enzymes, can also be targeted for acetylation, and many of these are important in chemoprotective cellular processes (103). However, due to their large number of targets, and inherent pleiotropic nature, the widespread use of HDAC inhibitors warrants a cautionary approach (65). Furthermore, HDAC inhibitor efficacy can be influenced by a variety of pre-existing factors, including current genome acetylation status, age, environmental exposures, lifestyle, and even underlying inflammation (65). Thus, a better understanding of the divergent and cell-type-specific effects of dietary HDAC inhibitors, and the identification of routes to improve their systemic bioavailability will be necessary before their therapeutic efficacy can be fully realized.

#### Chemopreventive potential of dietary modulators of ncRNAs

ncRNAs have been shown to regulate nearly all biological processes, and by silencing oncogenes and upregulating tumor suppressor gene expression both lncRNAs and miRNAs can contribute to cancer initiation, promotion, and progression. For example, the miRNA-34 family is significantly upregulated by the tumor suppressor *TP53*, and helps mediate cell cycle arrest and apoptosis by repressing targets such as cyclin D1 and BCL2 apoptosis regulator (115, 116). Likewise, the lncRNA *LOC285194*, is also regulated in a *TP53*-dependent manner, and displays tumor-suppressive functions (19). Contrarily, the lncRNA HOX transcript antisense RNA (*HOTAIR*) is upregulated in numerous types of cancers and is instead a driver of malignancy (117). Thus, utilization of dietary agents that can promote anticarcinogenic ncRNA expression, or repress their pro-oncogenic functions, is a desirable cancer-preventative approach. Research demonstrating the utility of dietary interventions to target lncRNAs is limiting, but extensive evidence exists supporting dietary-based miRNA targeting for cancer prevention (**Table 3**). Although the majority of research supporting this idea has been in vitro and in animal models, promising early-stage clinical trials are now under way.

**TABLE 3:** Chemopreventive regulation of miRNA by bioactive dietary compounds^1^

Bioactive component	Source	Target ncRNA	Anticancer effects	Type of cancer	Model system	Reference
All-*trans* retinoic acid	Vitamin A	miR-10a, 15a/16-1, 107, 223, Let-7a-3/let7	↓Invasiveness ↑Apoptosis	Leukemia, breast cancer	Leukemia patients and cell lines, human breast biopsies	125, 126
Apigenin	Fruits and vegetables	miR-138	↑Apoptosis ↓Tumorigenesis	Neuroblastoma	Cell lines, mouse xenografts	127
Butyrate	Soluble fiber	miR-17-92a cluster	↓Proliferation, ↑Apoptosis	Colon cancer	Healthy human subjects, cell lines	124, 128, 129
Canolol, 4-vinyl-2,6-dimethoxyphenol	Crude canola oil	miR-7	↓Inflammation, ↓Proliferation	Gastric cancer	Cell lines, human prostatectomies	130
Curcumin	Turmeric	miR-21, 22, 15-5, 20a, 27a, 34a/c, 101, 141, 200b, 200c, 203, 205, MEG3	↑Drug sensitivity ↓Proliferation ↓Invasiveness	T-cell lymphoma, pancreatic cancer, colon cancer, prostate cancer, bladder cancer	Cell lines, chicken embryo metastasis assays, mouse xenografts, human biopsies	131–136
Curcumin-difluorinated	Curcumin analog	miR-21, 34, 200, 210, 143, Let-7	↑Apoptosis ↓Angiogenesis	Pancreatic cancer, colon cancer	Cell lines, mouse orthotopic xenografts, human biopsies	137–140
Diallyl disulphide	Garlic	miR-34a	↓Proliferation ↓Metastasis	Breast cancer	Cell lines	141
1α,25-Dihydroxycholecalciferol	Vitamin D	miR-22, 98, 181a, 181b, 627	↓Proliferation ↓Invasiveness	Breast cancer, colon cancer, prostate cancer	Cell lines, mouse xenografts	142–145
3,3′-Diindolylmethane	Cruciferous vegetables	miR-21, 31, 34a, 130a, 146b, 377	↓Proliferation, ↑Apoptosis	Lung cancer, prostate cancer	Cell lines, human prostatectomies, mouse carcinogen- induced lung cancer	146, 147
Docosahexaenoic acid	Fish oil	miR-15b, 16, 21, 22, 107, 143, 145, 191, 324-5p	↑Apoptosis ↓Inflammation	Colon cancer, breast cancer, glioma	Cell lines, mouse xenografts, rat carcinogen-induced colon cancer	148–151
Ellagic acid	Pomegranate	miR-27a, 126, 155, 215, 224	↑Apoptosis ↓Proliferation ↓Inflammation	Breast cancer, colon cancer	Cell lines, ACI rats, rat carcinogen-induced colon cancer, human colorectal cancer patients	152–156
EGCG	Green tea	miR-16, 34a, 145, 200c, 449c-5p, Let 7b	↑Apoptosis ↓Proliferation	Colon cancer, lung cancer, melanoma	Cell lines, mouse xenografts, mouse carcinogen-induced lung cancer	157–160
Folic acid		miR-21, 16a, 34a, 122, 127, 200b	↓Apoptosis	Hepatocellular carcinoma, colorectal cancer	Methyl-deficient rats, human biopsies, human patients with adenomatous colon polyps	161–163
Genistein	Soy	miR-29a, 34a, 574-3p, 1256, *HOTAIR*	↓Proliferation, ↓Invasiveness ↑Apoptosis	Prostate cancer, melanoma	Cell lines, human biopsies	164–167
α-Mangostin	Mangosteen	miR-143	↑Apoptosis	Colon cancer	Cell lines	168
PEITC	Cruciferous vegetables	miR-194	↓Invasiveness	Prostate cancer	Cell lines	119
ω-3 (n–3) PUFAs	Fish oil, walnuts	miR-16, 19b, 21, 26b, 27b, 93, 203, 297a	↑Apoptosis ↓Proliferation ↓Angiogenesis	Colon cancer	Mouse xenografts, mouse and rat carcinogen-induced colon cancer	123, 148, 169, 170
Proanthocyanidins	Grape seed extract	miR-19a, 20a, 21, 104, 148, 196a, 205, Let-7a	↑Apoptosis ↓Proliferation ↓Inflammation	Colon cancer	Mouse carcinogen-induced colon cancer	120
Resveratrol	Stilbenes	miR-17, 21, 34c, 328	↑Apoptosis ↓Proliferation ↓Invasiveness	Prostate cancer, pancreatic cancer, colon cancer, osteosarcoma	Cell lines, mouse xenografts, human biopsies	171–175
α-Tocopherol	Vitamin E	miR-122, 125b	↓Inflammation	Normal rat liver	Vitamin E–deficient rats	176

1 EGCG, (–)-epigallocatechin 3-gallate; *HOTAIR*, HOX transcript antisense RNA; PEITC, phenethyl isothiocyanate

As mentioned above, PEITC is a breakdown product of glucosinolates, a group of bioactive sulfur-containing compounds abundant in cruciferous vegetables. PEITC has been shown to exert anticancer effects by influencing both DNA methylation and histone modifications, and more recently, miRNA (118). In prostate cancer cells PEITC treatment upregulates miR-194 expression, which subsequently decreases invasive capacity by targeting bone morphogenic protein 1 and downregulating the expression of matrix metalloproteinases (119). These findings suggest that PEITC treatment could be used to decrease tumor aggressiveness and prevent metastasis.

Ideal cancer preventative agents, however, would work at the initiation phase of cancer progression to prevent onset of the disease entirely. In a mouse model of sporadic colorectal cancer, dietary-delivered grape seed extract was able to protect against azoxymethane-induced colon tumorigenesis by decreasing both tumor development and overall tumor size (120). Mechanistic analyses revealed that grape seed extract modulated miRNA expression profiles, as well as miRNA processing machinery, and that this was associated with an overall repression in cytokine and inflammatory signaling (120). Importantly, the bioactive components of grape seed extract are also well tolerated in humans (121). This is intriguing because nonsteroidal anti-inflammatory drugs have demonstrated anticancer properties, but are associated with increased gastrointestinal side effects (122). Thus, the miRNA-mediated anti-inflammatory properties of grape seed extract in humans should be further investigated.

In another animal model of colorectal cancer, HT-29 colon cancer cells were injected in mice, which were then placed on either a control or an isoenergetic walnut-containing diet. Tumors of mice consuming the walnut-containing diet had significantly higher concentrations of ω-3 (n–3) fatty acids, which was associated with significantly decreased tumor size (123). These findings are quite exciting because the walnut amount in the animal diet was equivalent to a very achievable 2 servings/d for humans (123). It is important to note that the changes in miRNA expression induced by chronic walnut consumptions were very modest, even in a genetically homogeneous strain of mice on a controlled diet. Thus, measurable diet-induced changes in miRNA expression may be difficult to assess in a diverse human population, although their physiologic impact could be quite powerful.

For example, it has previously been established that resistant starches that get metabolized into SCFAs are protective against colorectal cancer, whereas high red meat intake is associated with an increased risk. Most of these protective effects are attributed to the powerful HDAC inhibitory properties of SCFAs, such as butyrate; but SCFAs have the capacity to influence miRNA expression as well. In a study of healthy human volunteers, dietary supplementation with butyrylated high-amylose maize starch was able to protect against the induction of oncogenic miRNAs in the rectal mucosa of people eating a diet high in red meat (124). Importantly, the intake of the resistant starch with high red meat intake also correlated with increased expression of the tumor suppressor gene phosphatase and tensin homolog (*PTEN*), and decreased cell proliferation in rectal biopsies of healthy patients compared with those consuming the high red meat diet alone (124). This study highlights the potential for the protective and preventative effects on dietary modulation of miRNA in cancer prevention.

Unfortunately, the chemopreventive effects of dietary compounds seen in vitro are not very frequently recapitulated in vivo. In a double-blind, randomized controlled clinical trial investigating the influence of pomegranate ellagic acid on miRNA expression in the normal and malignant tissues of colorectal cancer patients, the researchers noted only modest changes in miRNA expression (152). Furthermore, the majority of the observed differences in miRNA expression between normal and malignant tissues were largely attributable to the tissue removal process, casting doubt on the clinical relevance of miRNA expression changes (152). Thus, although miRNA-mediated changes in gene expression may have significant physiologic implications, the use of miRNA expression profiling may never find widespread clinical utility. Another area of increasing research interest with regards to miRNA is investigating the utility of dietary-derived miRNAs to influence gene expression and cancer risk, but results to date remain controversial (177, 178).

### Part 3: necessary precautions for diet-based chemopreventive strategies

As mentioned above, poor bioavailability of dietary-derived bioactive compounds may be a primary reason we have not been able to recapitulate the cancer preventative results of preclinical studies (179). For example, ellagic acid (which is found in foods such as walnuts, berries, and pomegranates) is only slightly absorbed, and is instead extensively metabolized within the gut microbiota to urolithins, of which urolithin A exhibits the most promising anti-inflammatory and anticarcinogenic properties (180). However, following ellagic acid ingestion, urolithin A production is dependent upon the gene expression, body weight, and even the gut microbial ecology of the individual (181, 182). Interestingly, individuals can be categorized into 3 distinct ellagitannin-metabolizing phenotypes, or “metabotypes,” and this metabotyping can be used to explain interindividual variability in the improvement of cardiovascular risk markers in individuals consuming pomegranate (183). Ellagic acid metabotype could not be used to explain interindividual variability in gene and miRNA expression changes in colorectal patients following pomegranate extract consumption however (152, 181). Thus, when investigating the cancer protective capacity of dietary compounds, it is necessary to consider the individual differences in metabolism and the physiologic achievability of effective concentrations of their biologically active metabolites. The translatability of the tissue/cell culture model being utilized to understanding epigenetic modulation by the diet should also be considered.

Moreover, it is worth noting that despite the promising results of laboratory studies and small-scale clinical trials, very few dietary intervention strategies have been shown to be effective cancer-preventative agents in human trials. Indeed, many trials have been touted as overwhelming failures (184). In the randomized, double-blinded, placebo-controlled α-tocopherol and β-carotene primary prevention trial, 20 mg β-carotene supplementation per day unexpectedly increased lung cancer incidence by 18% (185). Likewise, in the β-carotene and retinol efficacy trial, daily supplementation with a combination of 30 mg β-carotene and 25,000 IU retinol (vitamin A) increased the relative risk of lung cancer by nearly 28% (186). However, these studies were conducted in smokers or workers exposed to asbestos, and thus a diet × environmental effect cannot be ruled out as an explanation of these negative results. In a secondary endpoint analysis, 50 mg of α-tocopherol acetate per day was associated with a 45% decrease in prostate cancer incidence (187). Contrarily however, in the Selenium and Vitamin E Cancer Prevention Trial, daily supplementation with 400 mg of α-tocopheryl acetate significantly increased prostate cancer risk (188). The large differences in doses and vitamin E sources could potentially explain these conflicting findings, but a piece of data that is notably missing from both cohorts is the starting α-tocopherol status of the subjects, which could also have significantly affected the outcomes.

The failure of nutrient supplementation to effectively prevent cancer is likely multifactorial, but in hindsight we now recognize that nutrient-based prevention may not be effective in subjects with adequate nutritional status. The Linxian Nutritional Intervention Trial found that supplementation with a combination of α-tocopherol (50 mg), β-carotene (15 mg), and selenium (50 µg) protected against cancer incidence and mortality, but it was performed in a population with recognized low intakes of micronutrients and significant nutrient insufficiencies (189). Similarly, in the Nutritional Prevention of Cancer Study conducted in the eastern United States, selenium supplementation was found to be beneficial, but only in individuals with low baseline concentrations of serum selenium (190). Moreover, in the Selenium and Vitamin E Cancer Prevention Trial, daily supplementation with 400 mg α-tocopheryl in patients with adequate concentrations of plasma α-tocopherol actually decreased circulating concentrations of γ-tocopherol by 50% (191). Because γ-tocopherol is also suspected to play a significant role in prostate cancer prevention, this decrease has been implicated in the significant increase in prostate cancer risk that was observed (36). This point is further underscored by epidemiologic evidence that suggests that deficiencies in iron and zinc, as well as folate, and vitamins B-12, B-6, and C, can increase cancer risk (192). Thus, nutrient-based chemopreventive efforts are likely best geared towards correcting nutritional inadequacies.

In addition to nutritional status, proper timing of dietary interventions is critical to successful dietary-based chemopreventive efforts. Findings from the Dutch hunger winter famine, and more recent work investigating the impact of maternal obesity, clearly indicate that early-life exposures are integral risk factors for cancer development (60, 61). Moreover, animal studies have clearly illustrated the role of the maternal diet during pregnancy in the epigenetic modifications associated with cancer formation (193–195). Although lifelong diet-based interventions are not realistic, evidence suggests that dietary chemopreventive efforts can still be effective as long as supplementation begins before the establishment of precancerous lesions (36). For example, in the Linxian Nutritional Intervention Trial, the combination of α-tocopherol/β-carotene/selenium was protective against esophageal squamous cell carcinoma in subjects aged <55 y, but not in those aged >55 y (196). This was likely because some degree of dysplasia was probably already present in the older, at-risk population (197). Thus, it may be important to integrate cancer-screening processes into dietary chemopreventive approaches. Due to the inherent challenges of lifelong dietary and lifestyle interventions, it may also be necessary to only target high-risk groups that are the most likely to benefit from such behavioral modifications.

Yet even if we identify a target group that would most likely adhere to, and benefit from, a dietary chemoprevention strategy, the question then becomes how will we test the efficacy of the intervention? Unlike genetic markers, epigenetic biomarkers are confounded by numerous variables in addition to diet, such as age, environment, and lifestyle. Thus, to assess the efficacy of a dietary intervention on an epigenetic marker for cancer prevention it would first be necessary to identify a defined biomarker that is either always present or always absent in all noncancerous individuals, and that is not susceptible to environmental influences. To date, a single such biomarker has not been identified, but the utility of assessing epigenetic marks as a component of clinical screenings has been established.

Measurement of Septin 9 methylation is now a part of an FDA-approved screening panel for the detection of colon cancer (198). Likewise, lack of methylation within the promoter of the DNA repair enzyme *O*^6^-methylguanine-DNA methyltransferase can be used to predict treatment response in adult glioblastoma patients (199). It is also worth mentioning that in addition to next-generation sequencing to investigate ncRNA abundance, it is now possible to perform rapid unbiased analysis of the total DNA methylome, as well as large-scale profiling of histone modifications (200). There is, then, considerable hope for identifying chemopreventive epigenetic markers, but it will first be necessary to distinguish a “healthy” epigenetic pattern before the utility of epigenomic profiling can be realized.

## Conclusions

Given the long latency of most cancers, and the physiologic factors that are known to be critical during cancer development, early-stage lifestyle interventions will likely be key to successful dietary-based chemoprevention. This point is underscored by evidence indicating that dietary-based chemopreventive efforts are most efficacious in individuals in whom no early signs of cancer have been detected (36, 196, 197). Inherent difficulties associated with this strategy, however, are determining the appropriate treatment duration, and assessing treatment efficacy in asymptomatic individuals. However, recent studies describing the utility of an “epigenetic clock” that can be assessed to predict disease risk based on epigenetic age may provide guidance for identifying optimal timing for dietary-based epigenetic intervention strategies (201, 202). Furthermore, because patient compliance can be problematic in long-term diet intervention trials, it may be necessary to target those high-risk groups that are most likely to benefit from such a behavioral modification. Thus, regular cancer screenings, and patient education should also be integrated into the design of chemopreventive studies.

It may also be that there are stages of life, such as early development, in which certain regions of the genome are more vulnerable to epigenetic alterations. For example, in utero exposure to both dietary restriction and excess can result in lasting changes to DNA methylation, and these alterations are associated with increased disease susceptibility (60, 203). And although conceptually, epigenetic modifications are reversible, evidence now indicates that prolonged exposure to epigenetic aberrations may eventually lead to irreversible alterations (204). We must then understand both the functional consequences of epigenetic marks and the associated temporal relations between these marks before we can prescribe effective diet-based interventions. The use of new technologies, such CRISPR, that allow for the recruitment of specific epigenetic writers and targeted epigenetic modifications will likely prove invaluable for understanding the epigenetic control mechanisms that contribute to cancer etiology.

When investigating the chemopreventive efficacy of dietary agents it is also important to consider that because of extensive metabolic processes, dietary intake does not necessarily reflect tissue or tumor exposure to biologically active compounds. A chemopreventive dietary agent can only be effective if sufficient concentrations of the biologically active components actually reach target organs. Accordingly, measures to enhance bioavailability of the bioactive component, such as optimizing the dosing regimen, incorporating it into a drug-delivery system, or synthesizing more stable bioactive analogs, should be taken. In this regard, it may also be necessary to assess the metabolic phenotype of the individual as well. Particularly for those bioactive components which are extensively metabolized by the gut microbiota, as microbial metabolism can have a significant impact on host epigenetic programming (205) and carcinogenesis [reviewed in (206)].

Nutritional status can also influence the chemopreventive efficacy of dietary compounds. Currently, there is no evidence that individual nutrients can or will be able to be used as pharmaceutical chemopreventive agents, except for in individuals in whom that nutrient is lacking. Indeed, preventing deficiencies in nutrients, such as iron, zinc, folate, and vitamins B-6, B-12, and C, has been suggested to play an important role in cancer prevention (192). The chemopreventive effects of adequate vitamin and mineral statuses are largely attributed to the prevention of DNA damage, but recently iron deprivation was also linked to aberrant changes in histone acetylation and methylation (207). New evidence also suggests that vitamin C may help regulate hematopoietic stem cell function and protect against leukemia progression via DNA demethylation (208, 209). Vitamin C has also been shown to augment the effectiveness of the clinically used DNMT inhibitor, 5-azacytidine, which could have significant therapeutic implications (210, 211). These finding suggest that epigenetic modifications may be yet another means by which micronutrient availability affects cancer development, and warrant continued investigation.

However, because individual

diets contain a mixture of healthy and less healthful constituents that can contribute to the overall chemopreventive efficacy of a bioactive compound, we are likely better off focusing on the overall dietary pattern rather than on a specific dietary agent. Indeed, synergistic effects of dietary bioactive compounds have been noted (80, 212–214), and even pharmacologic epigenetic therapies are seldom used as single agents, but rather in combination with other chemotherapeutics (26). The augmented therapeutic efficacy of combinatorial epigenetic treatments further highlights the importance of considering the chemopreventive actions of a given dietary compound within the full diet, and in the context of an entire lifestyle. Undoubtedly, the promotion of a healthy lifestyle that includes regular physical activity, prevention of overweight and obesity, and abstaining from smoking would undoubtedly improve the chemopreventive efficacy of any single bioactive dietary component.

## Notes

Start-up funds from Oklahoma State University supported this work.

Author disclosures: MM and AS, no conflicts of interest.

Abbreviations used: DNMT, DNA methyltransferase; EGCG, (–)-epigallocatechin 3-gallate; HAT, histone acetyltransferase; HDAC, histone deacetylase; lncRNA, long noncoding RNA; miRNA, microRNA; ncRNA, noncoding RNA; PEITC, phenethyl isothiocyanate; RISC, RNA-induced silencing complex.
